# Physiological and molecular bases of the boron deficiency response in tomatoes

**DOI:** 10.1093/hr/uhad229

**Published:** 2023-11-08

**Authors:** Junjun Li, Huihui Fan, Qianqian Song, Lili Jing, Hao Yu, Ruishan Li, Ping Zhang, Fei Liu, Weimin Li, Liangliang Sun, Jin Xu

**Affiliations:** Shanxi Key Laboratory of Germplasm Resources Innovation and Utilization of Vegetable and Flower, College of Horticulture, Shanxi Agricultural University, Taigu 030801, China; Shanxi Key Laboratory of Germplasm Resources Innovation and Utilization of Vegetable and Flower, College of Horticulture, Shanxi Agricultural University, Taigu 030801, China; Shanxi Key Laboratory of Germplasm Resources Innovation and Utilization of Vegetable and Flower, College of Horticulture, Shanxi Agricultural University, Taigu 030801, China; Shanxi Key Laboratory of Germplasm Resources Innovation and Utilization of Vegetable and Flower, College of Horticulture, Shanxi Agricultural University, Taigu 030801, China; Shanxi Key Laboratory of Germplasm Resources Innovation and Utilization of Vegetable and Flower, College of Horticulture, Shanxi Agricultural University, Taigu 030801, China; Shanxi Key Laboratory of Germplasm Resources Innovation and Utilization of Vegetable and Flower, College of Horticulture, Shanxi Agricultural University, Taigu 030801, China; Shanxi Key Laboratory of Germplasm Resources Innovation and Utilization of Vegetable and Flower, College of Horticulture, Shanxi Agricultural University, Taigu 030801, China; Shanxi Key Laboratory of Germplasm Resources Innovation and Utilization of Vegetable and Flower, College of Horticulture, Shanxi Agricultural University, Taigu 030801, China; Shanxi Key Laboratory of Germplasm Resources Innovation and Utilization of Vegetable and Flower, College of Horticulture, Shanxi Agricultural University, Taigu 030801, China; Shanxi Key Laboratory of Germplasm Resources Innovation and Utilization of Vegetable and Flower, College of Horticulture, Shanxi Agricultural University, Taigu 030801, China; Shanxi Key Laboratory of Germplasm Resources Innovation and Utilization of Vegetable and Flower, College of Horticulture, Shanxi Agricultural University, Taigu 030801, China

## Abstract

Boron is an essential microelement for plant growth. Tomato is one of the most cultivated fruits and vegetables in the world, and boron deficiency severely inhibits its yield and quality. However, the mechanism of tomato in response to boron deficiency remains largely unclear. Here, we investigated the physiological and molecular bases of the boron deficiency response in hydroponically grown tomato seedlings. Boron deficiency repressed the expression of genes associated with nitrogen metabolism, while it induced the expression of genes related to the pentose phosphate pathway, thereby altering carbon flow to provide energy for plants to cope with stress. Boron deficiency increased the accumulation of copper, manganese and iron, thereby maintaining chlorophyll content and photosynthetic efficiency at the early stage of stress. In addition, boron deficiency downregulated the expression of genes involved in cell wall organization and reduced the contents of pectin and cellulose in roots, ultimately retarding root growth. Furthermore, boron deficiency markedly altered phytohormone levels and signaling pathways in roots. The contents of jasmonic acid, jasmonoy1-L-isoleucine, trans-zeatin riboside, abscisic acid, salicylic acid, and SA glucoside were decreased; in contrast, the contents of isopentenyladenine riboside and ethylene precursor 1-aminocyclopropane-1-carboxylic acid were increased in the roots of boron-deficient tomato plants. These results collectively indicate that tomato roots reprogram carbon/nitrogen metabolism, alter cell wall components and modulate phytohormone pathways to survive boron deficiency. This study provides a theoretical basis for further elucidating the adaptive mechanism of tomato in response to boron deficiency.

## Introduction

Boron (B) is an essential trace element that directly affects flowering and crop production [1, 2]. In plants, B deficiency has been reported in more than 130 countries or regions around the world [3]. Several studies have demonstrated that the concentration range between B deficiency and B toxicity is very narrow in plants, which complicates the application of B fertilizer in agricultural production [4]. Elucidating the physiological and molecular mechanisms of the B deficiency response and B accumulation in plants is of great significance for improving plant B utilization efficiency and cultivating B-efficient crop varieties.

B nutrition is involved in the whole plant life cycle, including leaf development, root system growth, bud development, and lateral branch formation [5]. B deficiency increased chlorophyll contents in spinach and cotton [6]; however, the underlying molecular mechanism remains unclear. Cell wall development is a major target of B nutrition in plants [7]. RGII (rhamnogalacturonan II) is a component of pectin. B can form a borate diester bond by binding with apiose residues in RGII, thereby covalently cross-linking two RGII monomers to form RGII-B-RGII complexes in cell walls [7]. More than 95% of RGII is present as a borate cross-linked dimer RGII-B-RGII in *Arabidopsis*; however, the loss-of-function *MUR1* (*GDP-D-MANNOSE-4,6-DEHYDRATASE 2*) mutant *mur1* exhibits normal amounts of RGII in leaf cell walls, but only half is present as RGII-B-RGII, thereby resulting in a dwarfed phenotype and abnormal leaf development [8]. Exogenous application of borate rescues the defect of *mur1*, indicating that plant growth depends on B-mediated pectic polysaccharide organization in the cell wall [8].

B is mainly absorbed by the root in the form of uncharged boric acid [9]. Previous studies indicated that boric acid constitutes the main, and possibly unique, mechanism of membrane transport of B through passive diffusion of lipid bilayers [9]. However, in the past two decades, much evidence has shown that, in addition to passive diffusion, B also enters plant cells through boric acid or borate channels, especially under B deficiency conditions [2]. *Arabidopsis NIP5;1* (*NOD26-like intrinsic protein 5;1*) encodes a member of the MIP (major intrinsic protein) family of aquaporins and was the first identified borate channel protein [9, 10]. NIP5;1 is localized at the root plasma membrane and can be induced by B depletion in plants [5]. *Arabidopsis* NIP6;1 shares 83.1% similarity with NIP5;1 and mediates B transfer from the xylem to the phloem in the leaf node region, thereby regulating B distribution in aboveground tissues [9]. In addition to these aquaporins, BOR1 (boron transporter 1) is another important type of B transporter protein in plants [10, 11]. *BOR1* is mainly expressed in root epidermis, endodermis and pericycle cells for xylem loading [11]. Under B depletion, a loss-of-function *bor1* mutant exhibits a severe growth-defective phenotype [11]. Overexpression of *BOR1* in tomato shows obvious apical dominance and higher biomass than wild-type plants under B deficiency [4]. BOR2 encodes a plasma membrane-localized B exporter and shares high sequence similarity with BOR1. *BOR2* is expressed in the epidermis of the root elongation zone and lateral root caps [12]. The *bor1 bor2* double mutant plants exhibit severe defects in cell wall structure and growth under B depletion [12].

Several studies have indicated that B deficiency inhibits plant growth and development through phytohormone signaling pathways [13, 14]. B deficiency represses auxin polar transport in roots, thereby resulting in auxin accumulation in root tips [14]. Exogenous application of PEO-IAA [α-(phenylethyl-2-oxo)-indoleacetic acid], a synthetic antagonist of auxin receptor TIR1 (transport inhibitor response 1), alleviates B deficiency-mediated growth inhibition in *Arabidopsis* roots [15]. B deficiency induces ethylene biosynthesis by upregulating the expression of *ACS11*, an ACC (1-aminocyclopropane-1-carboxylic acid) synthase gene. Increased ethylene production caused by B deficiency promotes auxin accumulation, ultimately resulting in primary root growth inhibition [14]. B deficiency induces ROS (reactive oxygen species) overaccumulation [16]. Exogenous application of AgNO_3_, an ethylene blocking agent, or AVG (aminoethoxy vinyl glycine), an ACS inhibitor, reduces ROS levels in B-deficient plants, thereby alleviating primary root growth inhibition, suggesting that B deficiency-induced ROS accumulation occurs in an ethylene-dependent manner [17]. JA (jasmonic acid) regulates the B deficiency response in plants by modulating cell wall structure and components through synergistic action with ethylene [17]. BR (brassinolide) is also involved in B-regulated cell wall structure and components in plants. Exogenous application of eBL (24-epibrassinolide), an important brassinosteroid (BR), alleviates B deficiency-mediated root growth inhibition in *Arabidopsis* [18]. In addition, cytokinin is also involved in modulating B deficiency-mediated cell division and differentiation in roots [19].

Tomato (*Solanum lycopersicum* L.) is one of the most important horticultural crops in global production and serves as a model crop for studying the molecular mechanisms of growth and development of fleshy fruits [20]. However, there are few reports on the mechanisms of the B deficiency response and B accumulation in tomatoes. In this study, we investigated the physiological and molecular mechanisms of the B deficiency response in tomato seedlings. These results provide insights for constructing a B nutrition regulation system in tomato cultivation.

## Results

### Physiological responses of tomato seedlings under B deficiency

**Figure 1:**
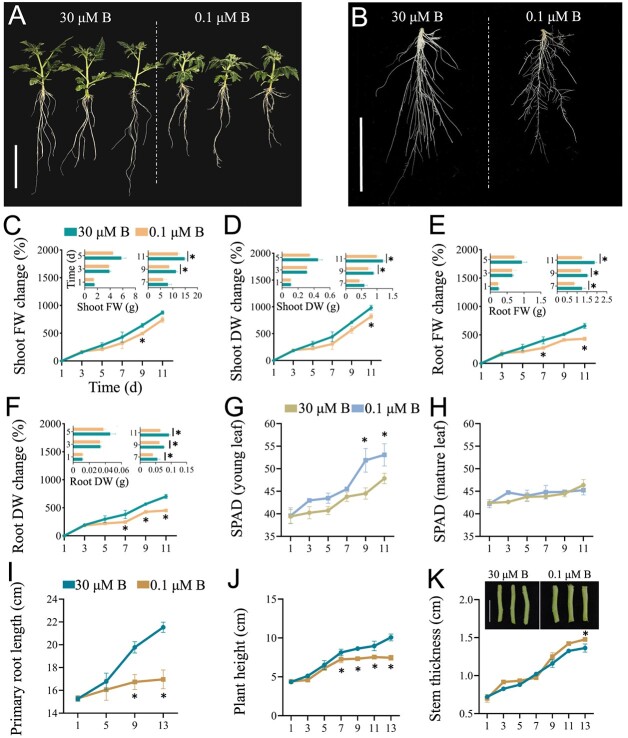
Boron deficiency inhibits tomato growth. **A** and **B,** Fourteen-day-old tomato seedlings were transferred to fresh Hoagland solution with sufficient B (30 μM B) or B-deficient conditions (0.1 μM B) for 13 d. Representative images show whole plants (**A**) and roots (**B**). Bar = 10 cm. **C–K**, Changes in shoot fresh weight (FW) (**C**), shoot dry weight (DW) (**D**), root FW (**E**) and root DW (**F**), SPAD values in young leaves (**G**) and mature leaves (**H**), primary root length (**I**), plant height (**J**) and stem thickness (**K**, bar = 2 cm). Values are given as the means ± SEs. Two-way analysis of variance (ANOVA), *P < 0.05.

Previous studies have shown that plant apical dominance, root growth, leaf expansion, flowering, and fruiting are inhibited when plants are subjected to B deficiency [21]. Consistent with these results, B deficiency markedly inhibited the growth of tomato seedlings (Fig. 1A and B). As shown in Fig. 1C–F, B deficiency reduced the fresh and dry weights of the shoots (reduced by 18.27% and 16.64%, respectively) and roots (reduced by 34.25% and 30.27%, respectively) of tomato seedlings after 11 d of treatment. Interestingly, B deficiency increased the chlorophyll content by 4.72% after 11 d of treatment in young leaves but not in mature leaves (Fig. 1E–H). B deficiency also inhibited PR length by 22.53% after 13 d of treatment (Fig. 1B and I). In addition, the plant height was reduced by 25.99% (Fig. 1J); in contrast, the stem thickness was increased by 6.67% after 13 d of B deficiency (Fig. 1K).

**Figure 2:**
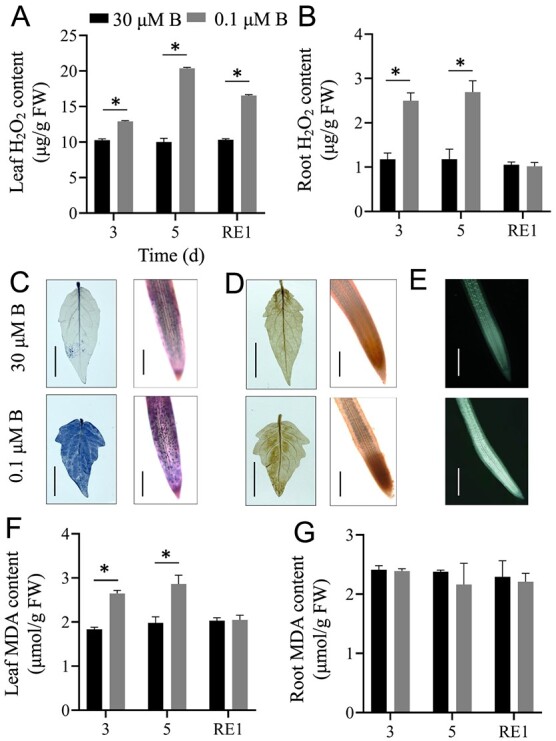
Boron deficiency induces oxidative damage in tomato seedlings. Fourteen-day-old tomato seedlings were transferred to fresh Hoagland solution with sufficient B (30 μM B) or B-deficient conditions (0.1 μM B) for 3 or 5 d. After 5 d of B deficiency treatment, the seedlings were transferred to normal Hoagland solution with sufficient B for recovery growth for 1 d (RE1). The H_2_O_2_ contents in the leaves (**A**) and roots (**B**) were determined. **C** and **D**, NBT staining (**C**) and DAB staining (**D**) showing the levels of O_2_^.-^ and H_2_O_2_ in the leaves (left, Bar = 1 cm) and roots (right, Bar = 300 μm) of tomato seedlings under sufficient B or B-deficient conditions, respectively. **E**, ROS levels were detected in the root tips of tomato seedlings under sufficient B or B-deficient conditions using a dichlorofluorescein diacetate (DCFH-DA) fluorescence probe. Bar = 500 μm. **F** and **G**, The MDA contents in the leaves (**F**) and roots (**G**) were determined. Values are given as the means ± SEs. Two-way analysis of variance (ANOVA), *P < 0.05.

Previous studies have demonstrated that B deficiency induces ROS bursts, thereby resulting in oxidative damage in plants [16, 22, 23]. We thus measured the H_2_O_2_ levels in tomato seedlings under B deficiency. As shown in Fig. 2, B deficiency increased H_2_O_2_ contents by 103.54% in tomato leaves and 128.37% in roots after 5 d of treatment (Fig. 2A and B). After 1 d of B resupply, the H_2_O_2_ content returned to normal levels in roots (Fig. 2B).

To further confirm the results, the leaves and roots were subjected to O_2_^.^-specific NBT staining (Fig. 2C) and H_2_O_2_-specific DAB staining (Fig. 2D). Both NBT and DAB staining showed that B deficiency induced the accumulation of H_2_O_2_ and O_2_^.-^ in tomato seedlings (Fig. 2C and D). Furthermore, ROS-specific DCFH-DA fluorescence probe staining also confirmed that B deficiency induced ROS accumulation in roots (Fig. 2E).

ROS overaccumulation can result in oxidative damage in plants. Subsequently, we measured MDA levels (a marker for the degree of membrane lipid oxidation) in tomato seedlings (Fig. 2F and G). B deficiency markedly increased leaf MDA contents, and after 1 d of B resupply, the MDA content returned to normal levels (Fig. 2F).

**Figure 3:**
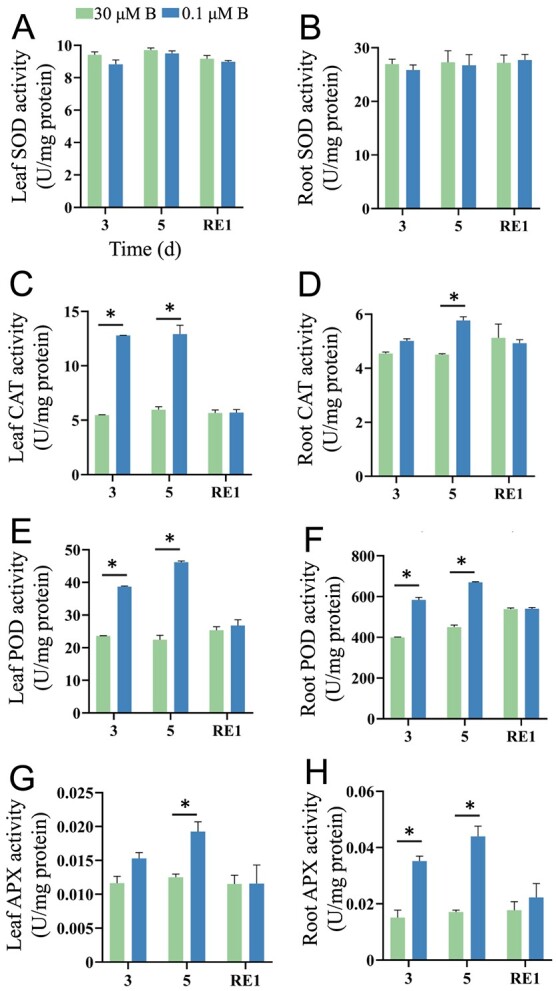
Boron deficiency affects antioxidative enzyme activities in tomato seedlings. Fourteen-day-old tomato seedlings were transferred to fresh Hoagland solution with sufficient B (30 μM B) or B-deficient conditions (0.1 μM B) for 3 or 5 d. After 5 d of B deficiency treatment, the seedlings were transferred to normal Hoagland solution with sufficient B for recovery growth for 1 d (RE1). The antioxidative enzyme activities were determined. **A**, Leaf superoxide dismutase (SOD) activity. **B**, Root SOD activity. **C**, Leaf catalase (CAT) activity. **D**, Root CAT activity. **E**, Leaf peroxidase (POD) activity. **F**, Root POD activity. **G**, Leaf ascorbate peroxidase (APX) activity. **H**, Root APX activity. Values are given as the means ± SEs. Two-way analysis of variance (ANOVA), *P < 0.05.

Next, we measured the activities of antioxidative enzymes in tomato seedlings under B deficiency (Fig. 3). B deficiency did not affect SOD activity in tomato seedlings (Fig. 3A and B); in contrast, the activities of CAT, POD, and APX were increased by 116.54%, 105.85%, and 53.99% in leaves and 28.2%, 48.91%, and 157.67% in roots after 5 d of treatment (Fig. 3C–H). After 1 d of B resupply, the activities of these antioxidative enzymes returned to normal levels (Fig. 3C–H).

**Figure 4:**
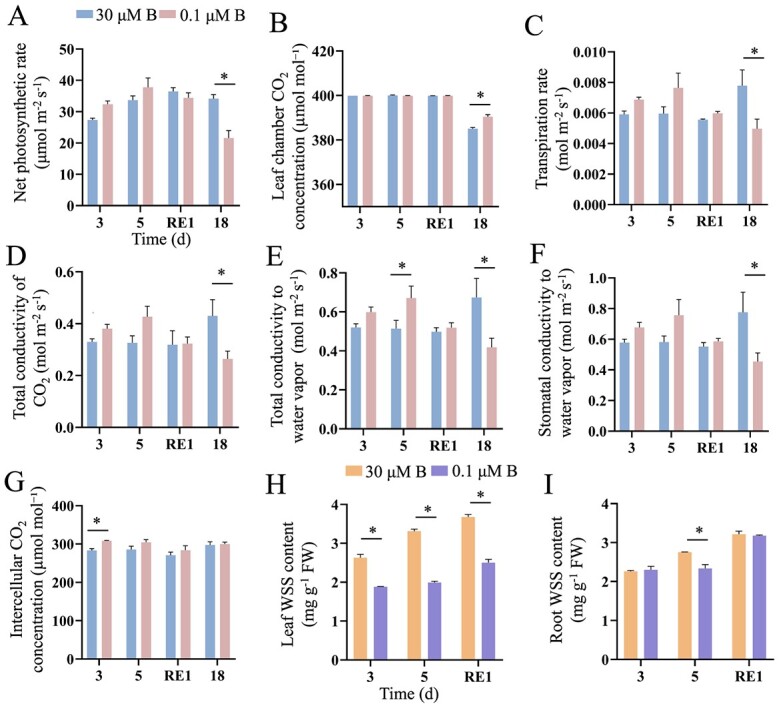
Boron deficiency affects photosynthesis efficiency in tomato seedlings. Fourteen-day-old tomato seedlings were transferred to fresh Hoagland solution with sufficient B (30 μM B) or B-deficient conditions (0.1 μM B) for 3 or 5 d. After 5 d of B deficiency treatment, the seedlings were transferred to normal Hoagland solution with sufficient B for recovery growth for 1 d (RE1) or continued to grow under B-deficient conditions for 18 d. The net photosynthetic rate (**A**), leaf chamber CO_2_ concentration (**B**), transpiration rate (**C**), total conductivity of CO_2_ (**D**), total conductivity to water vapor (**E**), stomatal conductivity to water vapor (**F**), intercellular CO_2_ concentration (**G**), and soluble sugar (WSS) contents in the leaves (**H**) and roots (**I**) were determined. Values are given as the means ± SEs, two-way analysis of variance (ANOVA), *P < 0.05.

B deficiency affects the transport of photosynthetic products and disrupts the absorption and utilization of nutrients [24]. We thus examined the effect of B deficiency on the photosynthetic efficiency in tomato seedlings (Fig. 4A–G). The intercellular CO_2_ concentration increased by 9% after 3 d of B deficiency, while the total conductivity to water vapor increased by 30.4% after 5 d of B deficiency. Both indexes returned to normal levels after 1 d of B resupply. In contrast to the short-term treatment, the long-term B deficiency (18 d) resulted in stronger changes in these photosynthetic indexes. The net photosynthetic rate, transpiration rate, total conductivity of CO_2_, total conductivity to water vapor, and stomatal conductivity to water vapor decreased by 36.75%, 36.26%, 38.52%, 38.07%, and 41.47%, respectively; in contrast, the leaf chamber CO_2_ concentration increased by 1.41% under long-term B deficiency. Sugar is a product of photosynthesis. B deficiency decreased the soluble sugar contents by 39.92% in tomato leaves and 14.99% in roots after 5 d of treatment (Fig. 4H and I). These results collectively indicated that B deficiency represses photosynthetic efficiency in tomato seedlings, ultimately retarding plant growth.

### High-throughput gene expression profiling analysis

**Figure 5:**
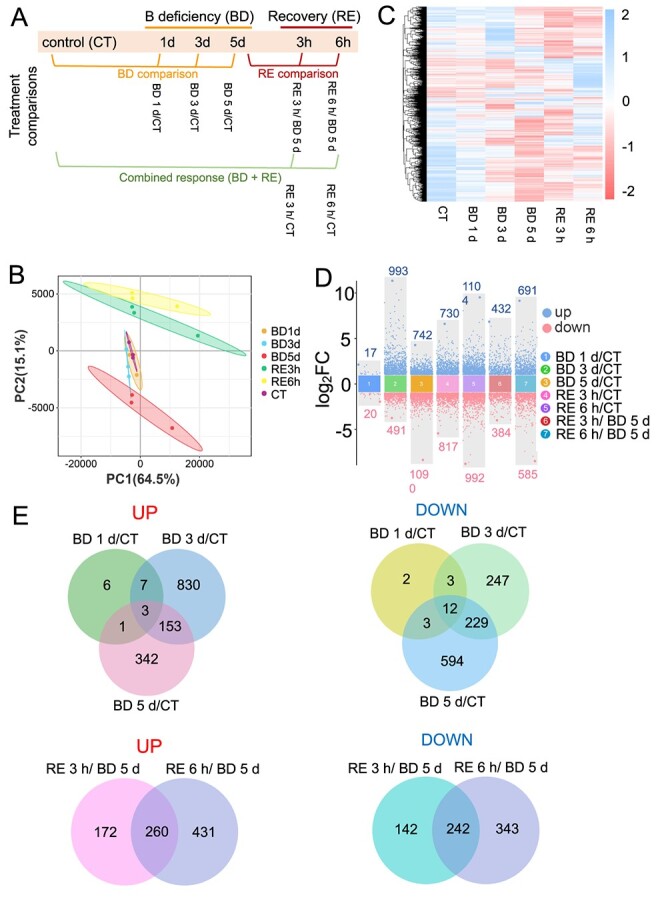
Transcriptome analysis of gene expression profiling in roots of tomato seedlings after boron deficiency (BD) or resupply after 5 d of BD treatment (RE). **A**, Overview of the experimental design of RNA-seq. Sequencing time points and treatment comparisons. **B**, Principal component analysis (PCA) of all genes. **C**, Clustering heatmap of all genes. **D**, Numbers of differentially expressed genes (DEGs) are shown in a multigroup difference scatter plot. **E**, The Venn diagram shows the intersection of DEGs for grouping comparison between different treatments. BD, B deficiency; CT, control; RE, B resupply after 5 d of BD treatment.

To better understand the molecular mechanism underlying B deficiency-mediated growth inhibition in tomato seedlings, we performed a transcriptome analysis to detect the DEGs (differentially expressed genes) in response to B deficiency using six treatment groups, including ‘CT’ (control), ‘BD 1 d’ (B deficiency for 1 d), ‘BD 3 d’, ‘BD 5 d’, ‘RE 3 h’ (recovery growth for 3 h in normal Hoagland solution after 5 d of B deficiency), and ‘RE 6 h’ (Fig. 5A). To analyse the DEGs in response to B deficiency and resupply, we divided the treatment comparisons into three categories: (i) B deficiency comparison (BD comparison), including ‘BD 1 d/CT’, ‘BD 3 d/CT’, and ‘BD 5 d/CT’ comparisons; (ii) recovery comparison (RE comparison), including ‘RE 3 h/BD 5d’ and ‘RE 6 h/BD 5d’ comparisons; and (iii) combined comparison (BD + RE), including ‘RE 3 h/CT’ and ‘RE 6 h/CT’ comparisons (Fig. 5A). A total of 133.54 Gb clean data with a Q30 base percentage of 94.31% or more was obtained from 18 samples by RNA-seq analysis. PCA (principal component analysis) clearly showed the separation of the first principal components among the treatments based on gene composition under B deficiency (Fig. 5B). Hierarchical clustering of all genes with FPKM (fragments per kilobase of exon model per million mapped fragments) >2 based on transcript similarity showed that there was good discrimination between the six treatment groups (Fig. 5C).

In the BD comparison, compared with CT, there were 37 (including 17 upregulated and 20 downregulated genes), 1484 (including 993 upregulated and 491 downregulated genes), and 1832 (including 742 upregulated and 1090 downregulated genes) DEGs in BD 1 d, BD 3 d, and BD 5 d, respectively. In the combined comparison (BD + RE), compared with CT, there were 1547 (including 730 upregulated and 817 downregulated genes) and 2096 (including 1104 upregulated and 992 downregulated genes) DEGs in RE 3 h and RE 6 h, respectively. In the RE comparison, compared with BD 5 d, there were 816 (including 432 upregulated and 384 downregulated genes) and 1276 (including 691 upregulated and 585 downregulated genes) DEGs in RE 3 h and RE 6 h, respectively (Fig. 5D).

Subsequently, the common and unique DEGs in the ‘BD 1 d/CT’, ‘BD 3 d/CT’, and ‘BD 5 d/CT’ comparisons were investigated using Venn diagram analysis. A total of three upregulated DEGs and 12 downregulated DEGs were coexpressed in the ‘BD 1 d/CT’, ‘BD 3 d/CT’, and ‘BD 5 d/CT’ comparisons. In addition, a total of 260 upregulated and 242 downregulated DEGs were coexpressed in the ‘RE 3 h/BD 5 d’ and ‘RE 6 h/BD 5 d’ comparisons (Fig. 5E). We randomly selected six genes to perform RT–qPCR analysis, and the results showed good consistency with the transcriptome data (Fig. S1, see online supplementary material).

### Weighted gene coexpression network analysis

**Figure 6:**
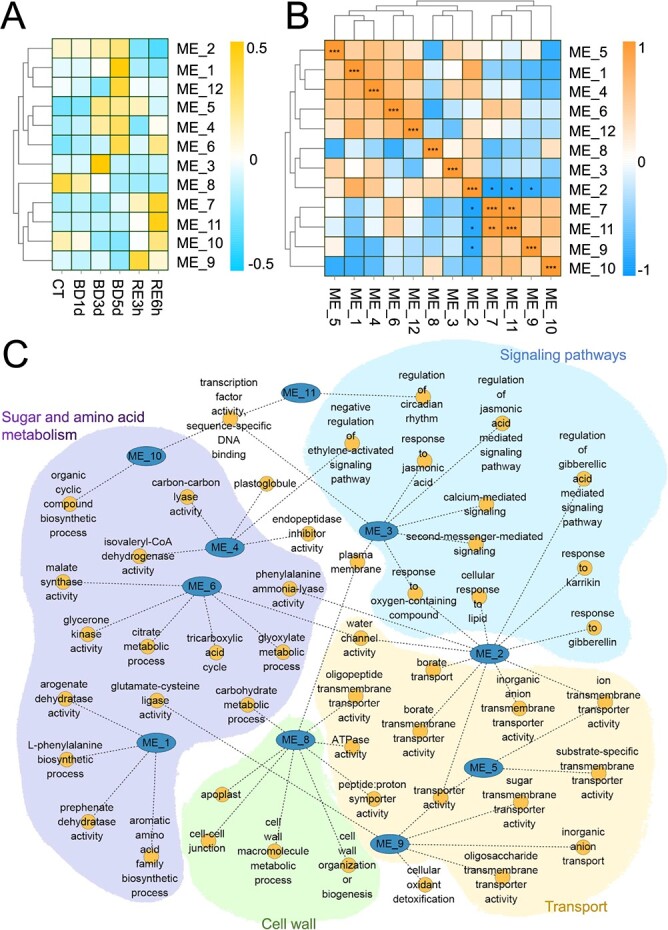
Gene Ontology (GO) analysis was performed on the transcriptome module obtained from weighted gene coexpression network analysis (WGCNA). **A**, Correlation analysis between the treatments and modules. The horizontal axis represents different treatments, while the vertical axis represents different modules divided by WGCNA. BD, B deficiency; CT, control; RE, B resupply after 5 d of BD treatment. **B**, Correlation analysis between modules. Asterisks indicate pairs with Pearson correlation coefficient (PCC) >0.75 or <−0.75 (*P < 0.05; **P < 0.01; ***P < 0.001). **C**, Integrated network of the transcript modules (false discovery rate <0.05).

We subsequently performed WGCNA (weighted gene coexpression network analysis) to investigate the regulatory network involved in B deficiency stress in tomato roots. A total of 12 gene modules (ME1-ME12) were obtained from WGCNA based on gene expression patterns and treatment groups (Fig. 6A). The correlation analysis heatmap between modules indicates that ME7 showed a strong positive correlation with ME11, while ME2 showed a negative correlation with ME7, ME9, and ME11 (Fig. 6B). Subsequently, a GO network diagram was constructed by analysing the significant modules and the GO pathways in these modules (Q value <0.05) (Fig. 6C). The network diagram was divided into four regions according to different biological functions, including sugar and amino acid metabolism, signaling pathways, cell wall component and structure, and ion transport. The ME1 and ME6 modules, which exhibited upregulated expression during B deficiency, mainly regulated sugar and amino acid metabolism. The ME2 module, which exhibited gradually upregulated expression with the extension of B deficiency and rapidly downregulated expression with B resupply, mainly regulated signaling pathways and ion transport. The ME3 module, which exhibited upregulated expression after 3 d of B deficiency, mainly regulated signaling pathways. The ME9 module, which exhibited downregulated expression during B deficiency but was rapidly upregulated with B resupply, mainly regulated ion transport. The ME8 module, which exhibited downregulated expression during B deficiency, mainly regulated cell wall components and structure and ion transport (Fig. 6C).

### B deficiency reprogrammed sugar and amino acid metabolism

**Figure 7:**
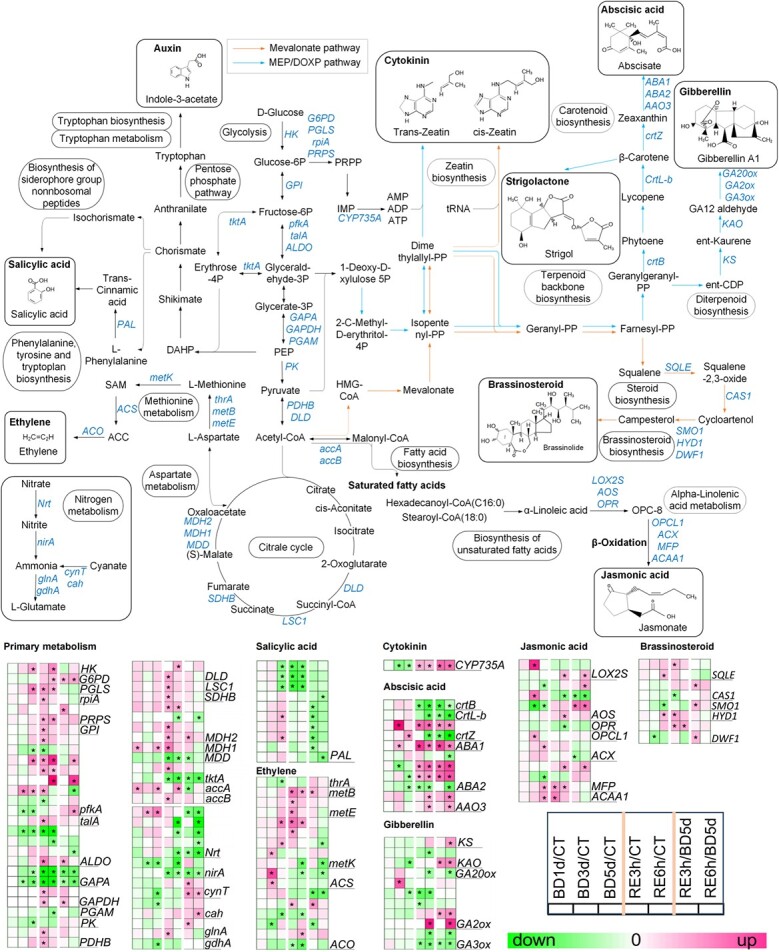
Boron deficiency affects the expression of genes associated with carbon/nitrogen metabolism pathways and phytohormone biosynthesis in tomato roots. BD, B deficiency; CT, control; RE, B resupply after 5 d of BD treatment. Heatmap analysis of gene expression patterns uses log_2_-fold change, and the asterisks in the heatmaps represent DEGs. The list of genes is shown in Table S1 (see online supplementary material).

The above results indicated that the soluble sugar contents were markedly decreased in B-deficient tomato seedlings (Fig. 4H and I), and our transcriptomic data showed that B deficiency modulated sugar and amino acid metabolism pathways (mainly in ME1 and ME6). We thus investigated the DEGs related to carbon/nitrogen metabolism (Fig. 7; Table S1, see online supplementary material).

#### Carbon metabolism pathway

In the BD comparison, compared with the control, the expression of one *HK* (*hexokinase*), one *PGLS* (*6-phosphogluconolactonase*), two *pfkA* (*6-phosphofructokinase A*), one *MDH1* (*malate dehydrogenase 1*), and one *accA* (*acetyl-CoA carboxylase carboxyl transferase subunit alpha*) gene was upregulated; in contrast, the expression of two *pfkA*, one *ALDO* (*fructose-bisphosphate aldolase*), two *GAPA* (*glyceraldehyde-3-phosphate dehydrogenase*), and one *PK* (*pyruvate kinase*) gene was downregulated under B deficiency. In the combined comparison (BD + RE), compared with the control, the expression of one *HK*, one *G6PD* (*glucose-6-phosphate 1-dehydrogenase*), one *PGLS*, one *rpiA* (*ribose 5-phosphate isomerase A*), one *PRPS* (*ribose-phosphate pyrophosphokinase*), one *GPI* (*glucose-6-phosphate isomerase*), five *pfkA*, one *talA* (*transaldolase*), one *ALDO* (*fructose-bisphosphate aldolase*), two *GAPDH* (*glyceraldehyde 3-phosphate dehydrogenase*), two *PDHB* (*pyruvate dehydrogenase E1 component subunit beta*), two *DLD* (*dihydrolipoyl dehydrogenase*), one *LSC1* (*succinyl-CoA synthetase alpha subunit*), one *SDHB* (*succinate dehydrogenase iron–sulfur subunit*), three *MDH2* (*malate dehydrogenase 2*), one *MDH1*, one *tktA* (*transketolase*), one *accA*, and one *accB* (*acetyl-CoA carboxylase biotin carboxyl carrier protein*) gene was upregulated; in contrast, the expression of two *pfkA*, two *ALDO*, two *GAPA*, one *PGAM* (*2,3-bisphosphoglycerate-dependent phosphoglycerate mutase*), one *MDH2*, one *MDD* (*malate dehydrogenase*), and one *tktA* gene was downregulated after B resupply. In addition, in the RE comparison, compared with the ‘BD 5 d’ group, the expression of one *G6PD*, one *PRPS*, one *ALDO*, one *GAPDH*, one *MDH2*, and three *pfkA* genes was upregulated; in contrast, the expression of one *pfkA*, one *ALDO*, two *GAPA*, one *MDH2*, one *MDD*, one *tktA*, and one *accA* gene was downregulated after 3 h and/or 6 h of B resupply (Fig. 7).

#### Nitrogen metabolism pathway

In the BD comparison, compared with the control, the expression of one *nirA* (*ferredoxin-nitrite reductase*), one *cynT* (*carbonic anhydrase*), one *cah* (*carbonic anhydrase*), and one *gdhA* (*glutamate dehydrogenase*) gene was downregulated; in contrast, the expression of one *Nrt* (*nitrate/nitrite transporter*) gene was upregulated under B deficiency. In the combined comparison (BD + RE), compared with the control, the expression of three *Nrt* and two *nirA* was downregulated; in contrast, the expression of one *glnA* (*glutamine synthetase*) gene was upregulated after B resupply. In the RE comparison, compared with the ‘BD 5 d’ group, the expression of two *cynT*, one *cah*, and one *glnA* gene was upregulated; in contrast, the expression of one *nirA* gene and all *Nrt* genes was downregulated after 3 h and/or 6 h of B resupply (Fig. 7).

### B deficiency disrupts phytohormone biosynthesis

**Figure 8:**
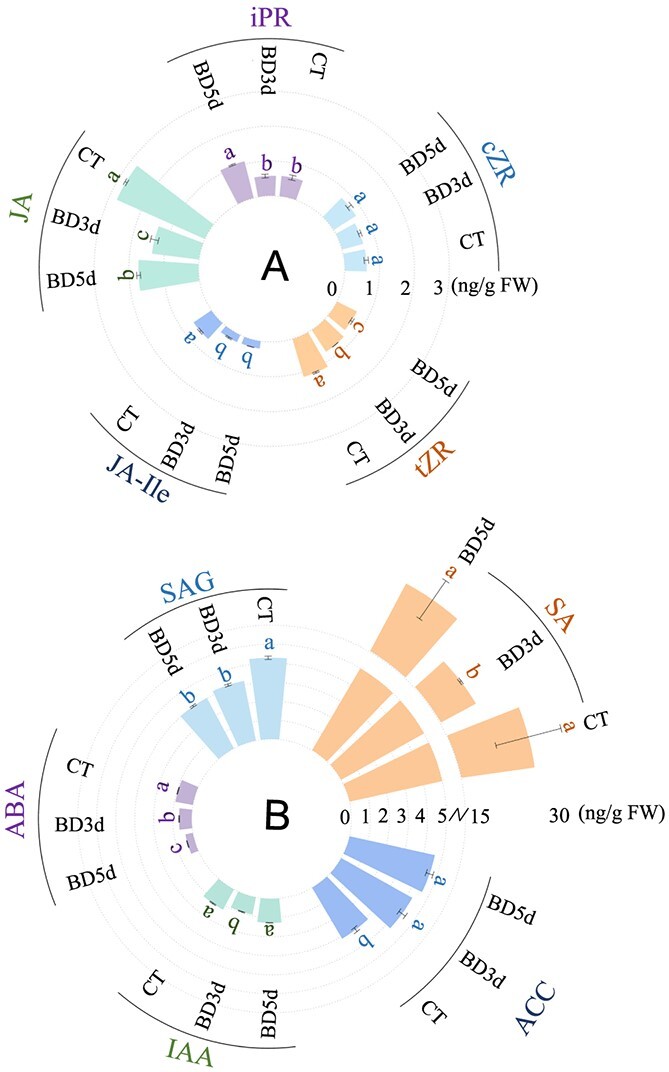
Boron deficiency alters phytohormone contents in the roots of tomato seedlings. Fourteen-day-old tomato seedlings were transferred to fresh Hoagland solution with sufficient B (30 μM B) or B-deficient conditions (0.1 μM B) for 3 or 5 d, and the contents of IAA, ABA, SAG, SA, and ACC were determined. Values are given as the means ± SEs, one-way analysis of variance (ANOVA). Different letters indicate differences between different treatments at a significance level of *P* < 0.05.

Phytohormones play critical roles in modulating growth and development in response to trace element levels [25]. Previous studies have demonstrated that phytohormones, including cytokinin, auxin, BR, JA, ABA, and ethylene, modulate B deficiency responses in plants [17, 19, 26–28]. However, whether and how B deficiency represses tomato root growth and development via phytohormone pathways remain largely unclear. To answer this question, we first examined phytohormone contents in tomato roots. As shown in Fig. 8, B deficiency decreased the contents of JA, JA-Ile, cytokinin tZR, ABA, SA, SAG, and IAA by 50.72%, 63.24%, 35.91%, 34.28%, 28.73%, 25.17%, and 23.56%, respectively, while it increased the ACC content by 37.65% after 3 d of B deficiency; meanwhile, it decreased the contents of JA, JA-Ile, tZR, ABA, and SAG by 37.71%, 65.41%, 43.65%, 56.69%, 29.37%, respectively, but increased the contents of ACC and cytokinin iPR by 47.70% and 84.73%, respectively, after 5 d of B deficiency.

Subsequently, we investigated DEGs associated with phytohormone synthesis (Fig. 7). In the BD comparison, compared with the control, the expression of the tZR biosynthesis-related gene *CYP735A* (*cytokinin trans-hydroxylase*) [29] was downregulated in the roots of B-deficient tomato seedlings, which was consistent with the decreased tZR level in tomato roots (Figs 7 and8A). B deficiency decreased the contents of JA and JA-Ile (Fig. 8A). Consistent with these results, the expression of two *AOS* (*hydroperoxide dehydratase*) and one *MFP* (*enoyl-CoA hydratase/3-hydroxyacyl-CoA dehydrogenase*) gene was downregulated under B deficiency (Fig. 7). B deficiency also reduced the contents of SA and SAG (Fig. 8B). Consistent with these results, the expression of three *PAL* (*phenylalanine ammonia-lyase*) genes related to SA biosynthesis was downregulated under B deficiency (Fig. 7).

The expression of most DEGs related to ethylene biosynthesis was upregulated under B deficiency, including two *ACS* (*1-aminocyclopropane-1-carboxylate synthase*) and three *S-adenosylmethionine synthetase* (*metK*). The upregulation of these ethylene biosynthesis genes was responsible for the increased root ACC level (Fig. 8B).

The expression of three ABA biosynthesis-related *ABA2* (*xanthoxin dehydrogenase*) genes was downregulated under B deficiency. Consistent with these results, B deficiency reduced ABA levels in roots (Figs 7 and8B).

In the BD comparison, compared with the control, the expression of one *KAO* (*ent-kaurenoic acid monooxygenase*) gene, which is related to GA (gibberellin) synthesis, was downregulated under B deficiency; however, in the RE comparison, compared with the ‘BD 5 d’ group, its expression was upregulated after B resupply. In addition, in the RE comparison, the expression of one *KS* (*ent-kaurene synthase*) gene, which is the second key gene in the GA synthesis pathway that can catalyze the synthesis of precursor substances to produce GA, was upregulated after B resupply compared with the ‘BD 5 d’ group. Moreover, compared with the ‘BD 5 d’ group, after 3 h and/or 6 h of B resupply, the expression of two *GA2ox* (*gibberellin 2beta-dioxygenase*) genes, which encode an enzyme that can reduce the level of bioactive GA levels in plants [30], was upregulated; in contrast, the expression of two *GA3ox* (*gibberellin 3beta-dioxygenase*) and one *GA20ox* (*gibberellin-44 dioxygenase*) genes, which are responsible for active GA biosynthesis in plants [31], was downregulated (Fig. 7).

B deficiency also altered the expression of genes related to BR biosynthesis. In the BD comparison, compared with the control, the expression of one *SQLE* (*squalene monooxygenase*) and one *HYD1* (*cholestenol delta-isomerase*) gene was upregulated; in contrast, the expression of one *SMO1* (*plant 4,4-dimethylsterol C-4alpha-methyl-monooxygenase*) and one *DWF1* (*delta24-sterol reductase*) gene was downregulated under B deficiency. In the combined comparison (BD + RE), compared with the control, the expression of one *SQLE*, one *HYD1,* and one *DWF1* was upregulated; in contrast, the expression of one *CAS1* (*cycloartenol synthase*) gene was downregulated after B resupply. In addition, in the RE comparison, compared with the ‘BD 5 d’ group, the expression of one *CAS1*, one *SMO1*, and one *DWF1* gene was upregulated after 3 h of B resupply (Fig. 7).

### B deficiency altered phytohormone signaling pathways

**Figure 9:**
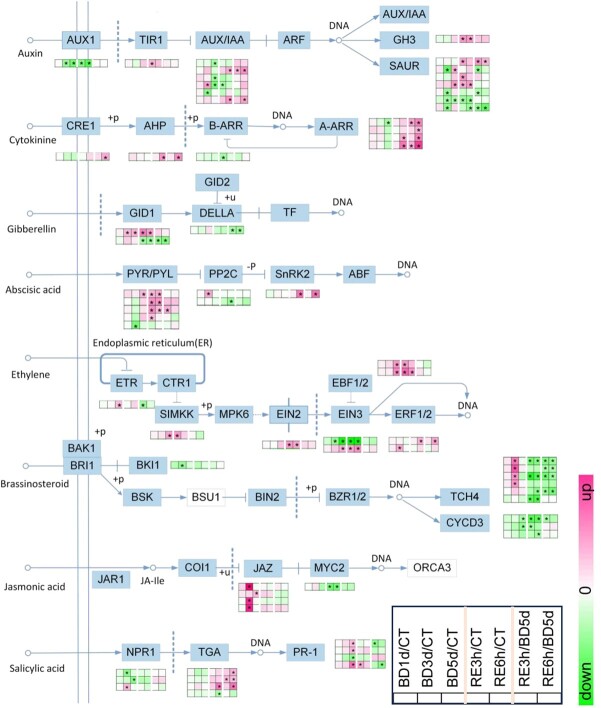
Boron deficiency affects the expression of genes associated with phytohormone signaling pathways in tomato roots. BD, B deficiency; CT, control; RE, B resupply after 5 d of BD treatment. Heatmap analysis of gene expression patterns uses log_2_-fold change, and the asterisks in the heatmaps represent DEGs. The list of genes is shown in Table S2 (see online supplementary material).

The above results showed that B deficiency altered phytohormone levels in tomato roots (Fig. 8). We thus investigated the changes in phytohormone signaling pathways (Fig. 9; Table S2, see online supplementary material). In the auxin pathway, compared with the control (BD comparison and/or combined comparison), the expression of the auxin influx carrier *AUX1* gene was downregulated under B deficiency, and its expression was not recovered after 6 h of B resupply. The expression of the auxin receptor *TIR1* gene was upregulated after B resupply. Early auxin response genes include three gene families, namely, *GH3* (*gretchen hagen 3*), *AUX/IAA* (*auxin/indoleacetic acid*), and *SAUR* (*small auxin up RNA*). In the BD comparison, compared with the control, the expression of two *SAUR* and one *AUX/IAA* gene was upregulated; in contrast, the expression of four *AUX/IAA* and four *SAUR* genes was downregulated under B deficiency. Moreover, the expression of two *AUX/IAA*, one *GH3* and three *SAUR* genes was upregulated, while the expression of one *AUX/IAA* and two *SAUR* genes was downregulated after B resupply (Fig. 9).

In the cytokinin pathway, compared with the control (BD comparison and combined comparison), the expression of one *A-ARR* (*type-A Arabidopsis response regulator*) gene was downregulated under B deficiency. Moreover, the expression of two *A-ARR* and one *AHP* (*Arabidopsis histidine phosphotransfer protein*) genes was upregulated; in contrast, the expression of one *B-ARR* (*type-B Arabidopsis response regulator*) gene was downregulated after B resupply. In the RE comparison, compared with the ‘BD 5 d’ group, the expression of one cytokinin receptor *CRE1* (*cytokinin response 1*) gene, one *AHP* gene and all *A-ARR* genes was upregulated after B resupply (Fig. 9).

In the GA pathway, compared with the control (BD comparison and combined comparison), the expression of one GA receptor *GID1* (*GA-insensitive DWARF1*) gene was upregulated under B deficiency and was not recovered after B resupply; in contrast, the expression of another *GID1* gene was downregulated after B resupply. In the RE comparison, compared with the ‘BD 5 d’ group, the expression of one *GID1* gene and *DELLA* gene was downregulated after B resupply (Fig. 9).

In the ABA pathway, compared with the control (BD comparison and combined comparison), the expression of one ABA receptor *PYR/PYL* (*pyrabactin resistance* (*PYR*)/*PYR-like*) and one *PP2C* (*protein phosphatase 2C*) gene was upregulated; in contrast, the expression of one *PYR/PYL* gene was downregulated under B deficiency. Moreover, the expression of four *PYR/PYL* and one *SnRK2* (*serine/threonine-protein kinase SRK2*) gene was upregulated; in contrast, one *PP2C* gene was downregulated after B resupply. In the RE comparison, compared with the ‘BD 5 d’ group, the expression of one *PYR/PYL* and one *SnRK2* gene was upregulated after B resupply (Fig. 9).

In the ethylene pathway, compared with the control (BD comparison and combined comparison), the expression of one *ETR* (*ethylene receptor*), one *SIMKK* (*mitogen-activated protein kinase kinase*), one *EIN3* (*ethylene-insensitive protein 3*), two *EBF1/2* (*EIN3-binding F-box protein 1 and 2*), and one *ERF1/2* (*ethylene response factor 1/2*) gene was upregulated; in contrast, the expression of one *EIN3* gene was downregulated under B deficiency. Moreover, the expression of one *SIMKK*, one *EIN2*, one *EIN3*, two *EBF1/2,* and one *ERF1/2* gene was upregulated; in contrast, one *EIN3* gene was downregulated after B resupply. In the RE comparison, compared with the ‘BD5d’ group, the expression of one *EBF1/2* gene was upregulated; in contrast, one *ETR* gene was downregulated after B resupply (Fig. 9).

In the BR pathway, compared with the control (BD comparison and combined comparison), the expression of four *TCH4* (*xyloglucan: xyloglucosyl transferase*) genes was upregulated; in contrast, the expression of one *BKI1* (*BRI1 kinase inhibitor 1*) and two *CYCD3* (*cyclin D3*) genes was downregulated under B deficiency. Moreover, the expression of four *TCH4* and three *CYCD3* genes was downregulated after B resupply. In the RE comparison, compared with the ‘BD 5 d’ group, the expression of four *TCH4* and one *CYCD3* genes was downregulated after B resupply (Fig. 9).

In the JA pathway, compared with the control (BD comparison and combined comparison), the expression of all *JAZ* (*jasmonate ZIM domain-containing protein*) genes was upregulated under B deficiency. However, the expression of the *MYC2* (*Myelocyto-Motosis2*) gene was downregulated after B resupply (Fig. 9).

In the SA pathway, compared with the control (BD comparison and combined comparison), the expression of one *proregulatory tein NPR1* gene and three *PR-1* (*pathogenesis-related protein 1*) genes was upregulated; in contrast, the expression of two *NPR1* and one *PR-1* gene was downregulated under B deficiency. Moreover, the expression of one *TGA* (*TGACG-binding factor*) and one *PR-1* gene was upregulated; in contrast, the expression of one *TGA* gene was downregulated after B resupply. In the RE comparison, compared with the ‘BD 5 d’ group, the expression of three *TGA* genes was upregulated; in contrast, the expression of two *PR-1* genes was downregulated after B resupply (Fig. 9).

### B deficiency increases metal ion accumulation in tomato seedlings

**Figure 10:**
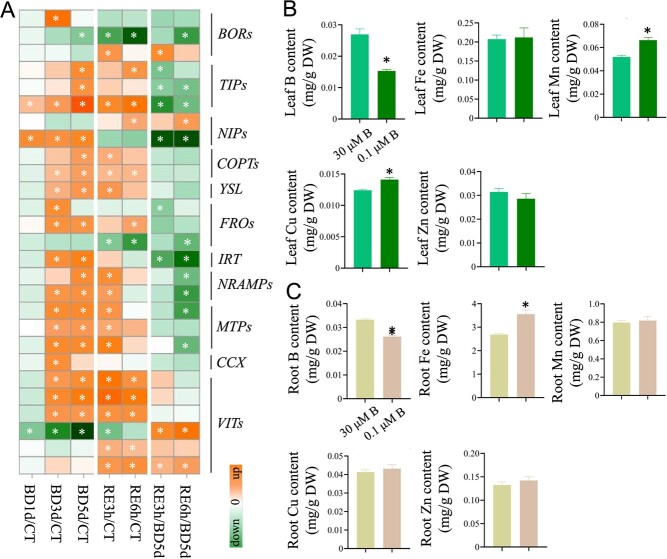
Boron deficiency affects micronutrient contents in tomato seedlings. **A**, Heatmap analysis of gene expression patterns using log_2_-fold change, and the asterisks in the heatmaps represent DEGs. BD, B deficiency; CT, control; RE, B resupply after 5 d of BD treatment. The list of genes is shown in Table S3 (BD, B deficiency;). **B** and **C**, Fourteen-day-old tomato seedlings were transferred to fresh Hoagland solution with sufficient B (30 μM B) or B-deficient conditions (0.1 μM B) for 15 d, and the micronutrient contents in the leaves (**B**) and roots (**C**) were determined. Values are given as the means ± SEs, Student’s *t* test, * *P* < 0.05.

GO analysis indicated that ion uptake and transmembrane transport were enriched (Fig. 6C). We thus investigated the DEGs involved in ion transmembrane transport in tomato roots. A total of 27 DEGs were identified, including three *TIP* (*tonoplast intrinsic protein*), three *BOR* (*boron transporter*), two *NIP* (*NOD26-like intrinsic protein*), two *COPT* (*copper transporter*), one *YSL* (*yellow stripe-like protein*), three *FRO* (*ferric reduction oxidase*), one *IRT* (*iron-regulated transporter*), three *MTP* (*metal tolerance protein*), two *NRAMP* (*natural resistance-associated macrophage protein*), one *CCX* (*calcium exchanger*), and six *VIT* (*vacuolar iron transporter*) (Fig. 10A; Table S3, see online supplementary material). Compared with the control (BD comparison and combined comparison), the expression of one *BOR*, three *TIP*, one *NIP*, two *COPT*, one *YSL*, two *FRO*, one *IRT*, two *NRAMP*, three *MTP*, one *CCX*, and three *VIT* genes was upregulated; in contrast, the expression of one *BOR* and one *VIT* gene was downregulated under B deficiency. Moreover, the expression of one *BOR*, two *TIP*, one *NIP*, two *COPT*, one *YSL*, one *FRO*, two *NRAMP*, three *MTP*, and five *VIT* genes was upregulated; in contrast, the expression of one *BOR*, one *FRO*, and one *VIT* gene was downregulated after B resupply. In the RE comparison, compared with the ‘BD 5 d’ group, the expression of one *BOR*, one *NIP*, and two *VIT* genes was upregulated; in contrast, the expression of one *BOR*, three *TIP*, one *NIP*, two *FRO*, one *IRT*, two *NRAMP*, and two *MTP* genes was downregulated after B resupply (Fig. 10A).

We next detected the contents of trace elements in tomato seedlings. B deficiency markedly reduced the B content by 43.26% and 21.36% in tomato leaves and roots, respectively, while it increased the Fe content by 32.26% in roots and the contents of Mn (manganese) and Cu (copper) by 27.87% and 13.71% in leaves (Fig. 10B and C).

### B deficiency altered cell wall components in tomato roots

**Figure 11:**
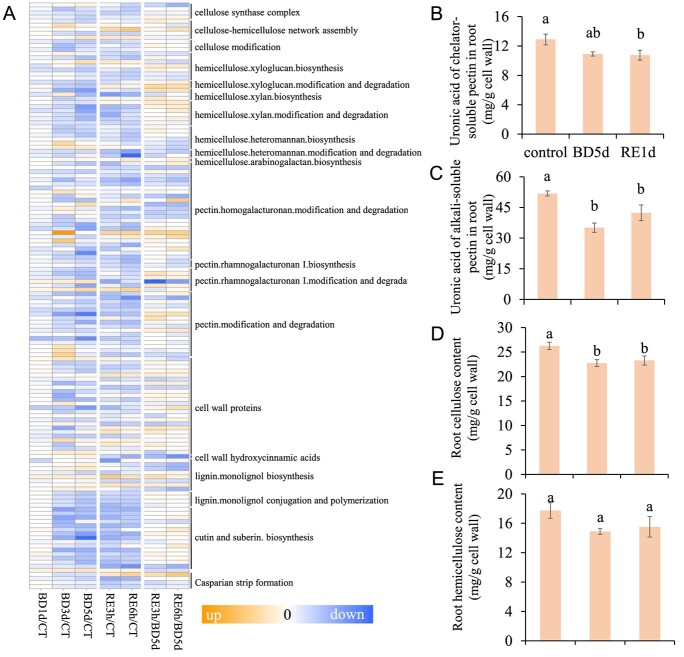
Boron deficiency alters cell wall components. **A**, Boron deficiency affects the expression of genes associated with cell wall biosynthesis and metabolism in tomato roots. BD, B deficiency;CT, control; RE, B resupply after 5 d of BD treatment. Heatmap analysis of gene expression patterns uses log_2_-fold change, and the asterisks in heatmaps represent DEGs. The list of genes is shown in Table S4 (see online supplementary material). **B**–**E**, Fourteen-day-old tomato seedlings were transferred to fresh Hoagland solution with sufficient B (30 μM B) or B-deficient conditions (0.1 μM B) for 5 d. After 5 d of B deficiency (BD 5 d) treatment, the seedlings were transferred to normal Hoagland solution with sufficient B for recovery growth for 1 d (RE1d). The contents of uronic acid of chelator-soluble pectin (**B**), uronic acid of alkali-soluble pectin (**C**), cellulose (**D**), and hemicellulose (**E**) in roots were detected. Values are given as the means ± SEs, one-way analysis of variance (ANOVA). Different letters indicate differences between different treatments at a significance level of *P* < 0.05.

The above results revealed that cell wall-associated GO pathways were enriched (Fig. 6C). We thus investigated the DEGs associated with cell wall metabolism processes, including cellulose and hemicellulose metabolism, pectin metabolism, cell wall proteins, lignin metabolism, and cutin and suberin biosynthesis (Fig. 11A; Table S4, see online supplementary material). The results showed that approximately 60% of the DEGs involved in cellulose metabolism, 80% of DEGs involved in hemicellulose metabolism, and 70% of DEGs involved in pectin metabolism were downregulated. Interestingly, the expression of these genes did not recover after B resupply.

Subsequently, we examined the contents of alkali-soluble pectin, chelator-soluble pectin, cellulose, and hemicellulose in roots (Fig. 11B–I). Consistent with the results of the transcriptome data, B deficiency reduced the alkali-soluble pectin content by 32.24% in tomato roots, while it gradually increased after B resupply (Fig. 11C). B deficiency also reduced the chelator-soluble pectin content by 15.4% in tomato roots (Fig. 11B), and ruthenium red staining further confirmed this result [32] (Fig. S2, see online supplementary material). Moreover, B deficiency reduced the cellulose content by 13.44% (Fig. 11D) but had no significant effect on the hemicellulose content (Fig. 11E).

## Discussion

B deficiency inhibited PR growth and decreased the biomass in tomato seedlings (Fig. 1A–F and I). Previous studies have shown that B deficiency increases stem thickness in cherry tomato and decreases plant height in cotton [6, 33]. Consistent with these results, we found that the stem diameter increased; in contrast, the plant height decreased in tomato seedlings after B deficiency (Fig. 1G, J and K). B deficiency induces ROS overaccumulation, thereby resulting in oxidative damage, as indicated by MDA contents, in plants [16]. Our results indicated that B deficiency increased ROS levels and MDA contents in tomato seedlings (Fig. 2). With the increases in ROS levels caused by B deficiency, the antioxidant enzyme activities in tomato seedlings significantly increased, including CAT, POD, and APX (Fig. 3C–H). This adaptive feedback mechanism enhances tomato tolerance to B deficiency stress. Previous studies indicated that B deficiency repressed photosynthesis efficiency and reduced sugar accumulation in citrus, cotton, tea, and sunflower [23, 34, 35]. Similar to these results, we found that long-term B deficiency reduced the photosynthetic rate and soluble sugar levels in tomato seedlings. These results indicated that B deficiency led to similar morphological and physiological changes in higher plants.

Maintaining carbon/nitrogen metabolism equilibrium is essential for plant growth and stress responses [36]. Lu *et al.* [37] found that B deficiency modulates the PPP (pentose phosphate pathway) in citrus. Our transcriptome data showed that the expression of most genes involved in the PPP, such as *GPI*, *pfkA*, and *talA*, was upregulated after B deficiency (Fig. 7), suggesting that B deficiency may induce more PPP precursors and enhance the PPP process in tomato roots. In addition, our transcriptome data showed that most DEGs involved in the EMP (Embden-Meyerhof pathway) and TCA (tricarboxylic acid cycle) were upregulated after B resupply (Fig. 7), indicating that B resupply recovered carbon metabolism in tomato roots.

B deficiency also affects nitrogen metabolism in plants [38]. B deficiency reduces nitrate uptake and represses nitrate reductase activity in tobacco and tomato [39, 40]. Consistent with these results, we found that the expression of genes related to nitrogen metabolism, such as one ferredoxin-nitrite reductase *nirA* and one glutamate dehydrogenase *gdhA*, was significantly downregulated after B deficiency in tomato roots (Fig. 7). Several studies have demonstrated that NIP subfamily aquaporins are also involved in organic nitrogen uptake in plants [41, 42]. NIP5;1 mediates urea uptake in *Arabidopsis* under B deficiency [41], while NIP2;1 mediates urea uptake in maize and cucumber [42, 43]. Our transcriptomic analysis revealed that B deficiency induces the expression of *NIP5;1-like* gene in tomato roots (Fig. 10A). Future research will elucidate whether this gene is involved in the uptake of organic nitrogen in tomato. Taken together, these results indicated that B deficiency led to metabolic reprogramming in tomato roots.

B homeostasis is regulated by BORs and aquaporins in plants. BOR1 is responsible for xylem loading of B, while the NIP subfamily aquaporin NIP5;1 mediates efficient root B uptake in *Arabidopsis* [11]. In addition, vacuolar membrane-localized TIP5;1 is also involved in B-mediated hypocotyl elongation in *Arabidopsis* [44]. In this study, we found that B deficiency induces the expression of *BOR* genes, *TIP* genes and one *NIP* gene in tomato roots (Fig. 10A). Furthermore, B deficiency also significantly induced the expression of genes involved in the uptake, transport, and accumulation of Fe in tomato roots, such as *FROs*, *IRTs*, *NARMPs*, and *VITs* (Fig. 10A). The Fe reductase FRO reduces Fe^3+^ to Fe^2+^ in the soil, and then Fe^2+^ is absorbed into roots by the Fe^2+^ transporter IRT1 [45]. NRAMPs are responsible for metal ion absorption and vacuolar compartmentalization, which are specific to plants [46]. VITs are also responsible for vacuolar compartmentalization of ferrous ions in roots [47]. The upregulated expression of these Fe transporters can explain the Fe accumulation in B-deficient tomato roots (Fig. 10A and C).

MTPs encode a class of metal transporters that maintain trace element dynamic equilibrium, such as Zn^2+^, Mn^2+^, and Fe^2+^, in plants [48]. COPTs are responsible for the transmembrane transport of Cu ions into the cytoplasm. In *Arabidopsis*, COPT3 and COPT5 are located in the inner membrane system, mainly transporting Cu ions in organelles to the cytoplasm, while plasma membrane-localized COPT1, COPT2, and COPT6 mainly mediate the absorption of external Cu ions [49]. YSLs are responsible for the long-distance transport of chelates formed by metal ions and NA (nicotianamine) [50]. B deficiency significantly upregulated the expression of *MTPs*, *COPTs*, and *YSL* in tomato roots (Fig. 10A). Consistent with these results, B deficiency induced the accumulation of Mn and Cu in leaves (Fig. 10B). Mn participates in photosynthesis and promotes chlorophyll biosynthesis [51]. Cu is involved in chlorophyll formation and maintains chlorophyll stability and photosynthetic efficiency in plants [52]. Indeed, the increased accumulation of Mn and Cu in leaves improved the chlorophyll contents in young leaves of tomato seedlings under B deficiency (Fig. 1G). These results are consistent with previous reports showing that B deficiency increased chlorophyll contents in *Arabidopsis* and cotton [53, 54] and suggested a general adaptation mechanism of plants in response to B deficiency.

The plant cell wall maintains cell morphology, enhances the mechanical strength of cells and provides important structural support for plant growth and development [7]. Previous studies have demonstrated that B can crosslink two RGII monomers in cell wall pectin to form an RGII-B-RGII dimer, thereby modulating cell wall structure [7]. B deficiency reduces pectin contents in *Arabidopsis*, *Poncirus trifoliate*, and *Beta vulgaris*, but interestingly, the changes in cellulose and hemicellulose are not significant [55, 56]. This may mean that cell wall B-binding sites are reduced and the pectin network is remodified. Similar to these results, we found that the contents of pectin and cellulose decreased in tomato roots after B deficiency (Fig. 11B–D), but there was no significant effect on hemicellulose (Fig. 11E). Furthermore, our transcriptome data showed that the expression of most DEGs related to pectin and cellulose metabolism was downregulated after B deficiency (Fig. 11A). A previous study indicated that pectin polysaccharides affect RGII-B-RGII compounds in the cell wall, thereby affecting the cell wall structure [8]. Therefore, the alterations in the cell wall components caused by B deficiency may affect the integrity of the cell wall, ultimately inhibiting root growth in tomato seedlings.

Phytohormones play a critical role in modulating the B deficiency response [14]. B deficiency inhibited the growth of tomato seedlings (Fig. 1) and reduced the contents of tZR, ABA, IAA, JA, and SA (Fig. 8). Our transcriptome data also showed that the expression of genes associated with the biosynthesis of IAA, cytokinin, GA, and ABA was downregulated after B deficiency (Fig. 7).

A previous study indicated that cytokinin levels were decreased in rapeseed seedlings under B deficiency [57], and the expression of cytokinin sensor *CRE1*/*WOL*/*AHK4* genes was downregulated under B deficiency in *Arabidopsis* [58]. Our transcriptomic data showed that the expression of the *CYP735A* gene, which encodes a cytokinin hydroxylase belonging to the cytochrome P450 monooxygenase family and regulates tZR content in plants through hydroxylation [59], was significantly downregulated after B deficiency (Fig. 7). Consistently, the tZR content in tomato roots significantly decreased after B deficiency (Fig. 8A). B deficiency also reduced the IAA content after 3 d of B deficiency; moreover, the expression of four *SAUR* and four *AUX/IAA* genes was downregulated (Fig. 9). Cytokinin and auxin antagonistically regulate plant morphogenesis [14, 60]. Under B deficiency, cytokinin inhibits *AUX1* expression, thereby altering auxin signaling in root tips; meanwhile, cytokinin also promotes ethylene biosynthesis by inducing *ACS11* expression, ultimately retarding root growth [15, 60]. Consistent with this result, we found that B deficiency upregulated the expression of 62.5% of ethylene biosynthesis-related DEGs and increased the contents of the ethylene synthesis precursor ACC (Figs 7 and8). Ethylene modulates cell wall metabolism [61]. Further study will elucidate how the B deficiency-mediated ethylene signaling pathway regulates cell wall components and structure. These results collectively indicated that B deficiency repressed the auxin and cytokinin pathways but induced the ethylene pathway, ultimately retarding root growth.

Previous studies indicated that B deficiency induces JA accumulation in plants by upregulating the expression of genes associated with JA biosynthesis under B deficiency [17]. Surprisingly, we found that JA levels were reduced in tomato roots after 3 d and 5 d of B deficiency (Fig. 8A). Moreover, our transcriptomic analysis revealed that the expression of six JA biosynthesis-related genes, including one *LOX2S*, one *AOS*, one *OPCL1*, one *ACX*, one *MFP,* and one *ACAA1* gene, was upregulated, whereas the expression of three JA biosynthesis-related genes, including two *AOS* and one *MFP*, was downregulated under B deficiency (Fig. 7). Zhou *et al.* [61] found that the contents of JA and JA-Ile decreased after 3 d of B deficiency but increased after 5 d of B deficiency in the roots of *Brassica napus*. Indeed, we also found that although B deficiency inhibited JA levels in tomato roots, the JA level in roots after 5 d of B deficiency was significantly higher than that after 3 d of B deficiency (Fig. 8A). Therefore, further research is needed to determine whether JA levels increase with the extension of B deficiency in tomato roots. In addition, previous studies have demonstrated that JA represses Fe uptake by downregulating the expression of *FIT/bHLH38/39/100/101* and subsequently inhibiting the expression of *IRT1* and *FRO2* [62, 63]. Therefore, under the early stage of B deficiency, the decrease in JA levels can explain the increase in Fe content in tomato roots (Fig. 10C).

In addition, several key genes involved in phytohormone biosynthesis exhibited opposite expression patterns under B deficiency and resupply, such as the ethylene biosynthesis gene *ACS3* and cytokinin biosynthesis gene *CYP735A2*, suggesting that these genes have a rapid response to B status, thereby modulating phytohormone levels and ultimately coordinating stress adaptation and growth in tomato seedlings. Future studies will elucidate phytohormone-mediated B deficiency adaptation in tomatoes by deciphering the regulatory network with the potential to improve the genetics of tomato varieties with efficient B utilization.

**Figure 12:**
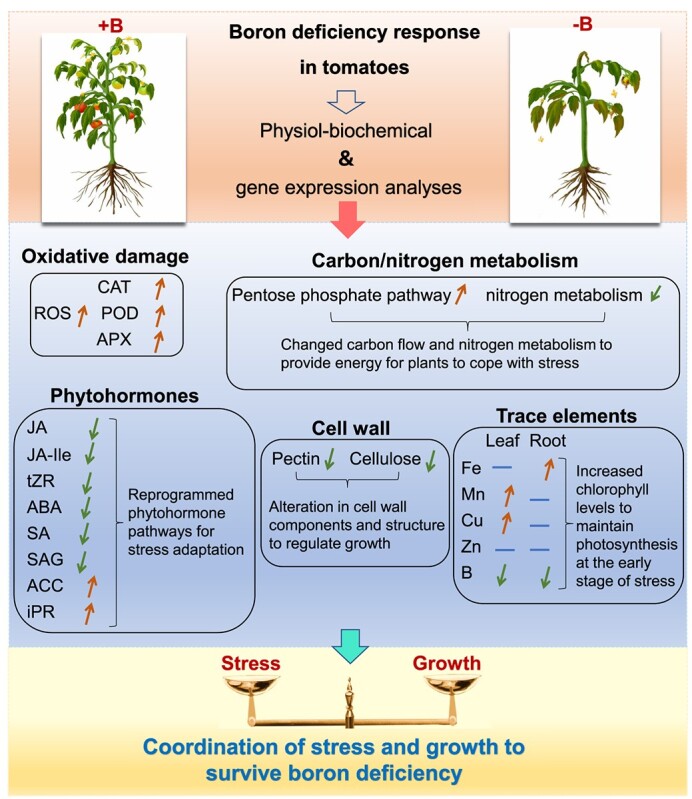
Overview of the boron deficiency response in tomatoes. Under boron deficiency, plants integrate antioxidant systems, metabolic homeostasis, micronutrient distribution, cell wall reconstruction, and phytohormone pathways to coordinate stress adaptation and growth. The upward or downward arrows indicate the increased and decreased levels, respectively.

In this study, we constructed a molecular mechanism model of the tomato B deficiency response through physiol-biochemical and transcriptomic analyses (Fig. 12). Our results revealed that (i) B deficiency induced oxidative damage, thereby retarding plant growth; (ii) B deficiency induced the expression of metal transporters and increased the accumulation of Cu, Mn, and Fe, thereby maintaining chlorophyll levels and photosynthetic efficiency at the early stage of stress; (iii) B deficiency upregulated the expression of PPP-related genes, while it downregulated the expression of genes related to nitrogen metabolism, ultimately leading to metabolic reprogramming; (iv) B deficiency downregulated the expression of genes involved in cell wall protein, cutin, and suberin biosynthesis, cellulose and hemicellulose metabolism, pectin metabolism and lignin metabolism in roots, thereby reducing the content of pectin and cellulose and remodifying the cell wall pectin network, ultimately inhibiting root growth; and (v) B deficiency reduced the levels of JA, JA-Ile, tZR, ABA, SA, and SAG but increased the levels of ACC and iPR, thus altering phytohormone signaling pathways. Taken together, this study provides us with a deeper understanding of the molecular mechanism of tomato in response to B deficiency.

## Materials and methods

### Plant material and growth conditions

Seeds of tomato (*S. lycopersicum* L.) *cv*. micro-Tom were sown on germination plates for 10 d. The uniform seedlings were transferred to fresh Hoagland solution for continuous growth for 14 d (24°C, 7200 lux, 16/8 h light/dark). Subsequently, 24-d-old seedlings were transferred to culture solution with sufficient B (30 μM B) or B-deficient conditions (0.1 μM B) for continued growth. The nutrient solution was replaced every 3 d.

### Determination of growth, chlorophyll content, and photosynthesis efficiency

The PR (primary root) length, plant height, and stem thickness were measured using ImageJ software (https://imagej.nih.gov/ij/) and Vernier caliper. The chlorophyll contents were determined using a SPAD-502 chlorophyll meter (Minolta Camera Co., Ltd., Tokyo, Japan). Photosynthesis efficiency was measured using a Li-6800 photosynthesis instrument (LI-COR, NE, USA). For specific operations, refer to Beijing Ligaotai Technology Co., Ltd. Li-6800 photosynthetic instrument user manual (https://www.ecotek.com.cn).

### Determination of antioxidative enzyme activities

The NBT method was used to determine the SOD (superoxide dismutase) activity as described by Agami and Mohamed [64], and the absorbance was detected at 560 nm. CAT (catalase) activity was determined as described by Du *et al.* [65], and the absorbance value was recorded every 15 s at 240 nm. The guaiacol method was used to determine POD (peroxidase) activity [66], and the absorbance value was recorded every 15 s at 470 nm. APX (ascorbate-peroxidase) activity was determined according to the methods of Elavarthi and Martin [67], and the absorbance values were recorded twice at a wavelength of 290 nm with an interval of 2 min.

### Determination of reactive oxygen species and malondialdehyde

The iodometric method was used to determine H_2_O_2_ contents [68], and the absorbance was recorded at 390 nm. MDA (malondialdehyde) contents were determined using the thiobarbituric acid method, and the absorbance was measured at 532 and 600 nm [69].

The ROS (reactive oxygen species) fluorescent probe DCFH-DA (2,7-dichlorofluorescein diacetate) (Beyotime, China) was used to visualize the level of endogenous ROS levels in root tips [70] and observed under a fluorescence microscope. For DAB (3-diaminobenzidine) staining, the leaves and roots were immersed in DAB-HCl (1 mg/ml, pH = 3.8) for 6–12 h and 30–60 min, respectively. For NBT (azuridine blue tetrazolium) staining, the leaves were immersed in NBT (0.5 mg/ml, pH = 7.8) for 3–6 h, and the roots were immersed in 0.5 mg/ml NBT (pH = 7.8) for 15–30 min. Images were taken using a digital camera and optical microscope.

### Determination of phytohormones

Fresh samples (100 mg) were ground in liquid nitrogen and then transferred to 1.5 mL of 50% acetonitrile extract containing 0.3 ng HABA (4′-hydroxyazobenzene-2-carboxylic acid) internal standard. After ultrasonication for approximately 3 min in an ice bath, the sample was centrifuged (13 000 rpm at 4°C) for 10 min, and the supernatant was then collected. The sample was purified using an Oasis HLB RP (Waters, USA) column, and the effluent was collected [71]. The effluent was dried with nitrogen at low temperature, resuspended in 30% acetonitrile, and then filtered with a 0.22 μM filter membrane. Phytohormones, including ABA (abscisic acid), JA, JA-Ile (Jasmonoy1-L-isoleucine), IAA (indole-3-acetic acid), cZR (cis-zeatin riboside), iPR (isopentenyladenine riboside), tZR (trans-zeatin riboside), ACC, SA (salicylic acid), and SAG (SA glucoside), were quantified using UHPLC–ESI–MS/MS (ultrahigh-performance liquid chromatography-electrospray ionization-tandem mass spectrometry) [71, 72].

### Determination of soluble sugar contents

Fresh sample (0.15 g) was ground into powder in liquid nitrogen and then transferred to 80% ethanol and placed in a water bath at 80°C for 30 min. After extracting 3–5 times, the sample was evaporated in a boiling water bath to a solution of less than 0.1 mL and reacted with anthrone reagent for colorimetry at 620 nm [73].

### RNA sequencing and quantitative reverse transcription PCR

Total RNA was extracted from the roots using TRlzol Reagent (Takara, Beijing, China). Sequencing libraries were constructed using the Hieff NGS Ultima Dual-mode mRNA Library Prep Kit for Illumina [Yeasen Biotechnology (Shanghai) Co., Ltd]. The libraries were sequenced using an Illumina NovaSeq platform. Differential expression analysis was performed using DESeq2 (adjusted *P* value <0.05, fold change ≥2) [74]. The *S. lycopersicum* SL4.0 genome was used as a reference genome. Reverse transcription of RNA was performed using a cDNA synthesis kit (gDNA Purge, Novoprotein, China). The specific primers for RT–qPCR (quantitative reverse transcription PCR) are shown in Table S5 (see online supplementary material).

### Determination of trace elements

Plant materials were digested using REVO microwave digestion and extraction equipment (LabTech, Beijing, China). The fresh samples were dried in an oven to constant weight. Approximately 0.1 g of sample powder was poured into a digestion tube containing 6 mL of 65–68% nitric acid for digestion. The contents of trace elements were determined using ICP–AES (inductively coupled plasma atomic emission spectroscopy; iCAP6300, Thermo Fisher Scientific, Waltham, USA). The international standard single element standard solution (B: GSB G 62003–90; Fe: GBW 08616; Mn: GBW (E) 080157; Cu: Gbw 08615; Zn: GBW 08620, China Institute of Metrology) was used to draw standard curves.

### Determination of pectin, cellulose, and hemicellulose

Approximately 3 g fresh plant samples were ground with liquid nitrogen, added to 30 mL of precooled ultrapure water, and then centrifuged at 5500 rpm for 12 min. The residue was washed three times with 80% ethanol, one time with a methanol/chloroform (1:1) mixture, and finally one time with 30 mL of acetone. The remaining insoluble residue was the crude cell wall [75]. The cell wall crude extract was freeze-dried using a freeze dryer (SCIENTZ, Ningbo, China).

Approximately 0.1 g of crude cell wall samples was immersed in 10 mL of imidazole solution (0.5 mol/L, pH = 7) and oscillated at 22°C for 1 d. The supernatant was collected by centrifugation, and extraction was repeated 2–3 times to obtain pectin I (chelated pectin). Pectin II (alkali-soluble pectin) was then extracted with 10 mL of 50 mM Na_2_CO_3_ containing 20 mM 1,2-cyclohexylenedinitrilotetraacetic acid (CDTA). The cell wall without pectin was extracted with 10 mL of 4 M KOH containing 0.1% NaBH_4_ at 22°C for 1 d and centrifuged, and the supernatant was collected to obtain hemicellulose. The precipitate was then washed with 8 mL of 0.03 M glacial acetic acid and alcohol. Cellulose was obtained by drying at 60°C until constant weight. The contents of pectin, hemicellulose, and cellulose were determined using the methods of Blumenkrantz and Asboe-Hansen [76].

### Pectin staining

Pectin staining was performed according to the ruthenium red (RR) staining method proposed by Durand *et al.* [32]. Briefly, approximately 2–3 cm of root tips were excised and dyed in 0.05% ruthenium red solution for 10–15 min and then washed with dH_2_O more than five times. The images were obtained using a fluorescence microscope (excitation at 359 nm and barrier at 461 nm).

### Statistical analysis

Experiments were repeated at least three times, with each replicate using six seedlings for biological replication. Statistical analysis was performed using SPSS (Statistical Package for Social Sciences) software (IBM SPSS Statistics 26), and GraphPad Prism 9 (San Diego, CA, USA) was used for data visualization analysis. ANOVA (analysis of variance) or Student’s *t* test was used to estimate the significance of differences (*P* < 0.05).

## Supplementary Material

Web_Material_uhad229

## Data Availability

The raw data of RNA-seq were archived at the SRA of NCBI under accession no. PRJNA910924.
